# Minimally Invasive Robotic Approach to Treating Cement Leakage Into the Psoas: A Case Report

**DOI:** 10.7759/cureus.98213

**Published:** 2025-12-01

**Authors:** Hiroto Makino, John Choi, Raghad Barri

**Affiliations:** 1 Orthopaedic Surgery, Faculty of Medicine, University of Toyama, Toyama, JPN; 2 Orthopaedic Surgery, Spine Ortho Clinic, Mornington, AUS

**Keywords:** cement-augmented screw, cement leakage, complication, robot-assisted spine surgery, s: lumbar spine disorders

## Abstract

A 78-year-old woman developed groin and thigh pain, numbness, and hip flexion weakness. Imaging revealed cement leakage from an L4 screw into the psoas, compressing a lumbar plexus branch. Using a spinal robotic system (ExcelsiusGPS, Globus Medical, Inc., Audubon, PA), a minimally invasive approach enabled precise incision planning, safe psoas dissection, and en bloc cement removal. Symptoms resolved immediately. Symptomatic intrapsoas cement leakage causing radiculopathy is rare, and robotic guidance contributed to a safe, accurate procedure.

## Introduction

Cement-augmented pedicle screws have become an established technique for improving fixation strength in patients with osteoporosis or compromised bone quality [1,2]. Although generally effective, this method carries a risk of cement leakage into segmental veins, the spinal canal, and paravertebral soft tissue [3]. Cement leakage into segmental veins is a risk for pulmonary embolism [4], and leakage into the spinal canal or foramen may cause nerve symptoms [5,6], whereas symptomatic leakage into paravertebral soft tissue has not been reported.

Here, we describe a unique case of radiculopathy caused by intrapsoas cement leakage from a cement-augmented pedicle screw, successfully treated through a minimally invasive, robot-assisted approach.

## Case presentation

**Figure 1:**
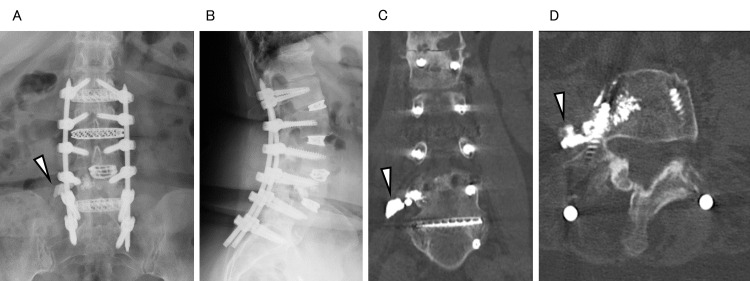
Preoperative images: (A) anteroposterior (AP) X-ray; (B) lateral X-ray; (C) coronal computed tomography (CT) image; (D) axial CT image (arrowheads indicating the leaked cement).

A 78-year-old woman presented with a three-month history of right thigh and groin pain, numbness, and hip flexion weakness. Her history included several lumbar surgeries, including an L3-4 transforaminal interbody fusion at another hospital seven years before admission, as well as L1-S1 lateral interbody fusion, posterior fusion, and bilateral sacroiliac joint fusion at our hospital two years before admission. She had also undergone bilateral total hip replacement two and three years before admission, respectively. Physical examination revealed hip flexion weakness of 4/5. Her pain and numbness coincided with the L3 or L4 dermatome. Radiographic and computed tomography (CT) images (Figure 1) showed cement leakage around a right L4 screw within the right psoas. Magnetic resonance imaging showed no other findings accounting for her symptoms. We chose to perform cement removal. For a minimally invasive procedure, we used a three-dimensional navigation robotic system with an ExcelsiusGPS (EGPS) (Globus Medical, Inc., Audubon, PA) to determine the optimal skin incision and guide psoas dissection.

**Figure 2:**
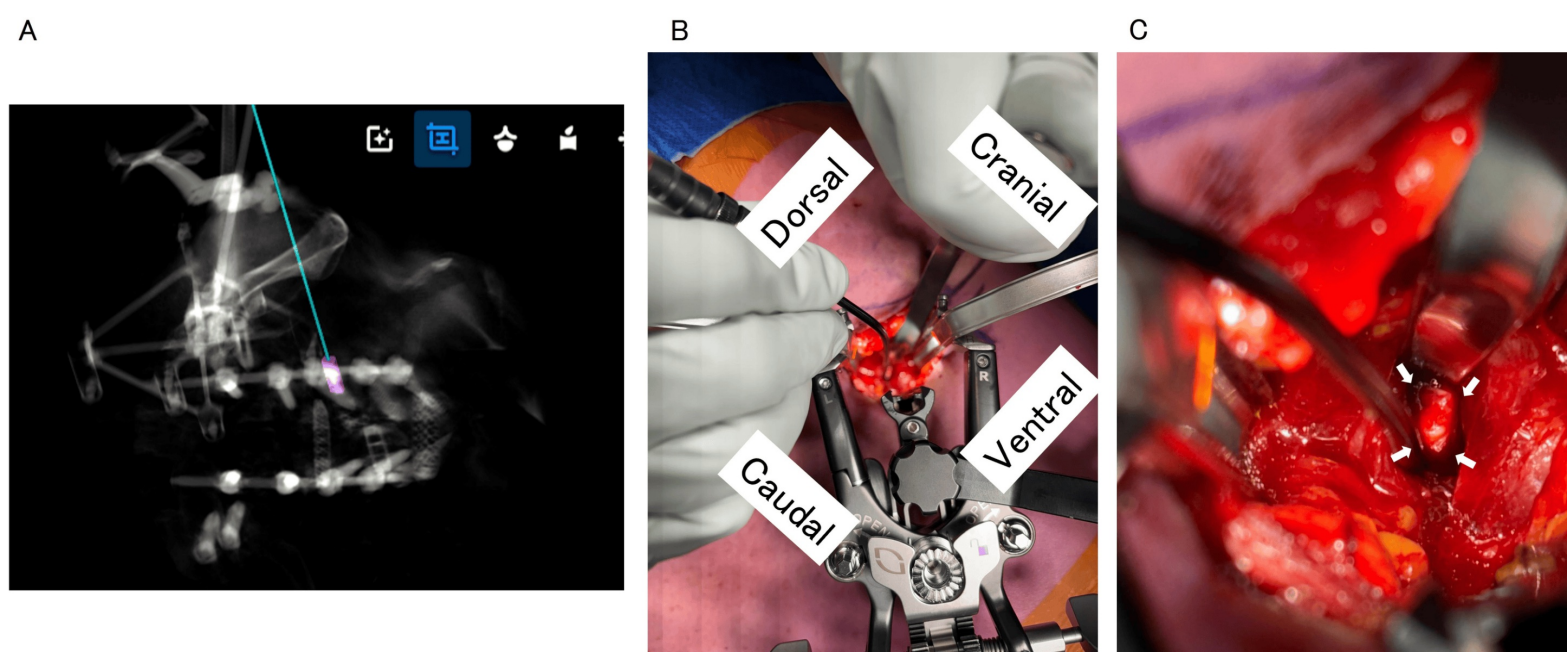
Intraoperative images. (A) Trajectory plan using EGPS. (B) A small skin incision and retractor placement. (C) A small dissection of the psoas and the leaked cement mass within the psoas. The branch of lumbar plexus passed directly above the cement mass (arrows, the cement mass). EGPS, ExcelsiusGPS

**Figure 3:**
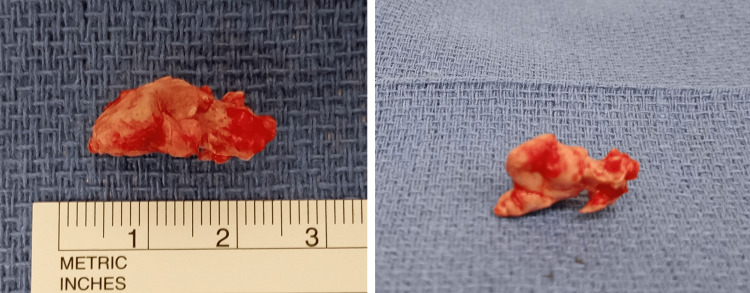
The removed cement mass. The leaked part was excised en bloc.

The patient was placed in the left decubitus position in the CT gantry. The reference was inserted into the posterior superior iliac spine. Intraoperative CT was acquired for navigation and robotic arm guidance, facilitating the virtual trajectory planning to target the leaked cement (Figure 2A). Under this guidance, an approximately 6 cm skin incision was made in the right abdominal wall using the robotic arm, and the psoas was exposed. Subsequently, A dilator was inserted via the robotic arm, and a retractor (MaXcess, Globus Medical, Inc.) was secured onto the psoas muscle. A navigation probe was used to precisely locate the cement (Figure 2B). The leaked cement was found compressing a branch of the lumbar plexus (Figure 2C) and was carefully removed in a single piece after blunt dissection of the affected nerve (Figure 3). The patient experienced immediate relief of groin and thigh pain and was discharged the following day. Three months after surgery, her hip flexion weakness remained unchanged.

## Discussion

Cement leakage is a complication of cement-augmented screws. Although its incidence varies across studies, Gao et al. [3] reported cement leakage occurring in 82% of patients and 35% of screws from a retrospective analysis of 202 cases involving 950 augmented pedicle screws. Another meta-analysis [7] reported a pooled risk of all cement leakage to be 21.8%, with symptomatic leakage occurring in only 1.2% of cases. The present case showed radiculopathy originating from the cement leakage within the psoas, which is the first case reported to our best knowledge. Considering these aspects, if nerve symptoms appear after surgery using cement-augmented screws, it is necessary to suspect cement leakage into the psoas as well as leakage into the spinal canal and foramen. Since there is a potential risk of injury to the lumbar plexus while dissecting the psoas [8], we applied EGPS to fix the location of the skin incision and psoas separation to mitigate neural complications. As a result, an improvement in preoperative symptoms was obtained without any complications. While the initial focus of robotic systems in spine surgery, including EGPS, was on accurate pedicle screw placement [9,10], other indications have been reported, including interbody cage placement and radiofrequency ablation in the treatment of aggressive sacral hemangiomas [11,12]. In these applications, robotic assistance provides a minimally invasive and highly precise means of accessing pathological sites. In our case, this advantage was useful in obtaining a good outcome. Robotic-assisted spine surgery offers a viable approach for managing atypical pathologies, such as the present extremely unusual case.

## Conclusions

This case highlights an exceptionally rare instance of radiculopathy caused by intrapsoas cement leakage from a cement-augmented pedicle screw. Precise localization and minimally invasive removal of the leaked cement were achieved with the assistance of EGPS, resulting in immediate symptom resolution without complications.
